# Long term impacts of endozoochory and salinity on germination of wetland plants after entering simulated seed banks

**DOI:** 10.3389/fpls.2023.1275622

**Published:** 2023-10-31

**Authors:** José L. Espinar, Jordi Figuerola, Andy J. Green

**Affiliations:** ^1^ Tragsatec, Seville, Spain; ^2^ Department of Conservation Biology and Global Change, Estación Biológica de Doñana, Consejo Superior de Investigaciones Científicas (CSIC), Seville, Spain

**Keywords:** Anatidae, dormancy, endozoochory, germination, salinity, seed banks, seed dispersal, waterbirds

## Abstract

Migratory waterbirds disperse a broad range of angiosperms by endozoochory (seed dispersal via gut passage), especially plants in coastal wetlands. However, there is no previous information about the capacity of seeds to remain in the seed bank after waterbird endozoochory, and very little about how wetland salinity can influence the effect of gut passage on germination. We collected seeds of *Juncus subulatus* (Juncaceae), *Bolboschoenus maritimus*, and *Schoenoplectus litoralis* (Cyperaceae) from Doñana marshes in Spain. All three species are considered to have physiological dormancy. After gut passage following ingestion by ducks, seeds were stored in darkness in solutions with six different conductivities (1, 2, 4, 8, 16, and 32 dSm^-1^), for periods of 1, 6, or 12 months to simulate presence in a seed bank. After storage, 1800 seeds of each plant species assigned to these treatments were subjected to germination tests in demineralized water, together with 1800 control seeds that had not been ingested before storage. All species germinated readily after storage, with or without gut passage beforehand. Storage time and salinity both had important effects on germinability and time to germination, which differed between control and ingested seeds, and between plant species. After ≥6 months, germinability of Cyperaceae was enhanced by gut passage (≤25% higher than control seeds) at some salinities. Only *J. subulatus* showed consistently lower germinability after passage (≤30%). Only *B. maritimus* showed consistently slower germination after passage (≤33%). Salinity effects were more complex after passage, but increasing salinity did not generally have a negative impact on germination of ingested seeds. When compared to additional seeds that had not been stored before germination tests, storage reduced germinability in *J. subulatus* (≤39% reduction), but increased it in *B. maritimus* (≤17%) and *S. litoralis* (≤46%). Seeds dispersed by waterbird endozoochory may be easily incorporated into wetland seed banks, where they can remain halotolerant and delay germination until conditions become suitable. This can benefit wetland plants by increasing rates of long-distance dispersal, gene flow, and establishment of new populations. Avian gut passage can have positive and species-specific effects on germination in plants with persistent seed banks and/or physiological dormancy.

## Introduction

Vertebrates are key vectors for seed dispersal, and provide greater dispersal distances than abiotic mechanisms, including wind (Bullock et al., 2017). The dominant paradigm in the literature assumes that only fleshy-fruited plants disperse regularly by endozoochory, i.e. the internal transport of propagules within the animal gut (Costea et al., 2019; Green et al., 2022). Hence, such plants are typically assigned to an “endozoochory syndrome”, whereas all other angiosperms are assigned to a range of other syndromes according to the interpretation of diaspore morphology. For example, despite early recognition of their regular dispersal by migratory waterbirds (Ridley, 1930; de Vlaming and Proctor, 1968), sedges (Cyperaceae) and rushes (Juncaceae) are variously assigned to abiotic syndromes such as hydrochory, anemochory, or barochory (Julve, 1998; Vargas et al., 2023). As a result, the role of endozoochory in seed dispersal has largely been overlooked for such plants (Green et al., 2022). The endozoochory of plants lacking a fleshy fruit can be considered “non-classical endozoochory” (Green et al., 2019).

Like many other plants with dry fruits, sedges and rushes are regularly dispersed between habitat patches such as wetlands inside the guts of migratory waterbirds, including ducks, geese, shorebirds, or gulls (Martín-Vélez et al., 2021a; Green et al., 2023; Lovas-Kiss et al., 2023a; Urgyán et al., 2023). Their seeds can be just as well adapted to survive gut passage as can those inside fleshy-fruits (de Vlaming and Proctor, 1968; Costea et al., 2019). Although many waterbirds have muscular gizzards containing grit that can break up seeds, experimental feeding studies have shown seeds of Cyperaceae, Juncaceae and other families to have high rates of seed survival, and to retain germinability during gut passage (Figuerola et al., 2010; Lovas-Kiss et al., 2020; Lovas-Kiss et al., 2023b; van Leeuwen et al., 2023). Modelling studies show seeds are readily dispersed by endozoochory over hundreds (or even thousands) of km during migratory flights, or over tens of km during daily movements outside migratory periods (Viana et al., 2013; Kleyheeg et al., 2017; Martín-Vélez et al., 2021b; Lovas-Kiss et al., 2023a). Field studies have confirmed the presence of seeds in the guts of birds during migratory flights (Fridriksson and Sigurdsson, 1968; Viana et al., 2016).

Seed dispersal by avian vectors may be central to metapopulation dynamics and gene flow in wetland plant populations. It can be vital for dispersal between hydrological catchments, for colonization of new habitats created by human activities, and to allow plants to keep pace with climate change (Green et al., 2023; Lovas-Kiss et al., 2023a; Nuñez et al., 2023). However, there is almost no research on how the potential establishment of plants after dispersal is influenced by endozoochory by waterbirds. Research on pondweeds (Figuerola and Green, 2004; Figuerola et al., 2005) showed that gut passage by ducks can accelerate germination and growth in the field or in mesocosms, although this may have a cost of increasing exposure to herbivory. There is far more research into establishment success following classical endozoochory (i.e. by frugivores, Schupp et al., 2017; Spennemann, 2020). Fleshy-fruited plants generally lack a persistent seed bank (Hulme, 2002), so their establishment must occur soon after a seed dispersal event.

In contrast, sedges, rushes, and other wetland plants typically form persistent seed banks that provide resilience to environmental variation, e.g. from annual and seasonal variation in depth or salinity (Clevering, 1995; Leck and Schutz, 2005; Nielsen et al., 2018), and which are regularly replenished by seed dispersal events (Polo-Ávila et al., 2019; Infante-Izquierdo et al., 2023). Salinity can have an important immediate effect on germination of wetland plants, even for halotolerant plants (Nielsen et al., 2003; Hroudova et al., 2014; Muñoz-Rodríguez et al., 2017; Castillo et al., 2021). The seed storage conditions in the soil seed bank (flooding and salinity) may also determine the subsequent seed response when conditions later become suitable for germination (Espinar et al., 2005a; Muñoz-Rodríguez et al., 2017; Castillo et al., 2021). In addition, the dormancy strategies of a given plant species are also expected to determine the germination response to environmental variables (Clevering, 1995), as well as to gut passage (Soltani et al., 2018).

A key question therefore, as yet unresolved, is if seeds dispersed by waterbirds to a new site have the potential to survive for weeks or months in a seed bank until conditions become suitable for germination. Seeds that have undergone endozoochory may often have to compete with others dispersed only locally by abiotic means such as hydrochory (Soomers et al., 2013; Carthey et al., 2016; Tomowski et al., 2023), so it is important to separate the effects of gut passage and time spent in a seed bank on their germinability and germination speed. How subsequent germination is influenced by seed bank salinity is another important question. The salinity gradient is central to the composition of plant communities in coastal wetlands (Muñoz-Rodríguez et al., 2017), and ongoing salinization of freshwaters is a major threat to biodiversity, and an important topic for further research (Cunillera-Montcusí et al., 2022). When seeds of sedges and rushes are placed for germination without first entering a seed bank, the effect of avian gut passage on seed germinability and germination time has already been found to depend on salinity (Espinar et al., 2004; Espinar et al., 2006).

In this study, we test the ability of two sedges (sea club-rush *Bolboschoenus maritimus* (L.) Palla, and shore club-rush *Schoenoplectus litoralis* Scharader) and a rush (Somerset rush *Juncus subulatus* Forsskal) to persist in seed banks for up to a year between endozoochory and germination. These helophytes occur in brackish to saline coastal wetlands in the Mediterranean region and elsewhere, which have intermittent flooding and continuous variation in depth and salinity (Espinar et al., 2005b). These species have previously been shown to have high rates of seed survival and germinability immediately after passage through ducks (Espinar et al., 2004; Espinar et al., 2006; Urgyán et al., 2023). Here, we compare seeds that have undergone gut passage and those which have not, after simulating their presence in the sediments of an inundated wetland of variable salinity. We consider how gut passage interacts with the time spent in a simulated seed bank, and its salinity, in determining the germinability and germination time for the three plant species. Given that all three species are considered to have physiological dormancy and not physical dormancy (Baskin and Baskin, 2004), our initial hypothesis was that gut passage would invariably reduce seed germinability and prolong their germination time, when compared to control seeds.

## Methods

### Study area and species

The three plant species studied, *J. subulatus*, *Bolboschoenus* (*Scirpus*) *maritimus*, and *Schoenoplectus* (*Scirpus*) *litoralis*, are rhizomatous emergent macrophytes, and are the major components of the perennial vegetation in the seasonal marsh within Doñana National Park, which lies within the delta of the Guadalquivir river in south-west Spain (37° 00’ N, 06° 38’ W, Espinar et al., 2005b; Lumbierres et al., 2017; Green et al., 2018). This is a senescent marsh largely isolated from tidal influence, and subject to seasonal flooding under the effect of precipitation, and storm and groundwater discharge via several streams (which has been reduced by pumping for agriculture, Camacho et al., 2022). There is a general trend for increasing salinity in the marsh due to a reduction of freshwater inputs and increased temperatures (Green et al., 2017; Paredes et al., 2021). There is a Mediterranean climate and a marked seasonality in water availability, with a clear differentiation between a dry season (June–November) and a wet one (October–April). *Juncus subulatus* inhabits shallow brackish waters in coastal areas of the Mediterranean and Irano-Turacic regions. The Cyperaceae *Schoenoplectus litoralis* and *B. maritimus* are widely distributed in shallow brackish water bodies of Europe, Asia and Africa. *B. maritimus* has a wider latitudinal range and also occurs in the Americas (Valdés et al., 2007; POWO, 2023). In the study area, these species have a persistent and high density seed bank, with seeds distributed at different soil depths and at least up to 16-20 cm (Espinar et al., 2005b). Viable seed density (estimated by germination and recovery tests), depends on species identity and soil depth, with peak values in top soil of around 3500 seeds/m^2^ for *S. litoralis*, 2900 seeds/m^2^ for *J. subulatus*, and 2200 seeds/m^2^ for *B. maritimus* (Espinar et al., 2005b).

The Doñana wetlands represent the most important wintering site for migratory Anatidae (ducks and geese) in Europe, and are also of importance for a range of other waterbirds (Rendon et al., 2008; Green et al., 2018), many of which ingest Cyperaceae and Juncaceae seeds (Martín-Vélez et al., 2021a; Almeida et al., 2022).

### Seed collection and pre-treatment

During August 2001, for each of the studied species ten sites holding populations were selected at random from an area of 6,000 ha in the southern part of the marshes of Doñana National Park (details in Espinar et al., 2004). From each site, the inflorescences were collected at random from 60 shoots separated from each other by at least 10 m. Achenes (hereafter “seeds”) were separated from the spikes, and different populations were mixed to produce a homogeneous sample representative of the study area. The seeds were initially stored dry in darkness at 4°C (after Clevering, 1995) for seven months, until the experiment began. These stratification conditions would be expected to break dormancy, at least for a fraction of the seeds, and therefore increase germinability when seeds were placed at higher temperatures at a later date (Clevering, 1995).

### Seed gut passage

In March of 2002, 20 semi-captive mallards *Anas platyrhynchos* (10 of each sex) were placed in individual cages with a mesh floor and given unlimited access to food (broken rice) and drinking water. This experiment was conducted at ‘‘La Cañada de los Pajaros” wildlife centre in Sevilla province, and some of the seeds recovered were used in the related studies of Espinar et al. (2004; Espinar et al., 2006). This experiment was carried out several years before legislation requiring specific bioethical permits was passed in Spain, but used similar protocols to more recent experiments approved with current legislation (Lovas-Kiss et al., 2020; Peralta-Sánchez et al., 2023). Removable metal trays were placed under the cages to recover any droppings. The next morning, approximately 500 seeds of *S. litoralis* and another 500 seeds of *J. subulatus* were force-fed to each bird. The next day, faeces were collected from the trays and transported in plastic bags to the laboratory. The mallards were kept in a large pen until a few days later, when the process was repeated with the same individuals. In this case, each bird was fed with 500 seeds of *B. maritimus*. The day after, the birds were returned to large ponds in the wildlife centre and faeces were collected from the trays as before. In the laboratory, faeces were washed in a 300 µm sieve, and intact seeds were separated. Recovered seeds were subsampled from those ingested by different ducks so that each of the 20 individuals contributed equally to the experimental seeds. Individual ducks vary in the proportion of seeds destroyed during digestion (Figuerola et al., 2010; Lovas-Kiss et al., 2020), and this procedure prevented those individual ducks which destroyed fewer seeds from contributing relatively more to those used in our experiments.

### Exposure time and salinity in storage, simulating seed banks

Saline solutions for seed storage were prepared by adding different amounts of sea salt (dried to 110°C for 24 h) to demineralized water to produce the desired salinity. Six different solutions (ranging from 1 to 20 g L^-1^) were used, with electrical conductivities (EC) of 1, 2, 4, 8, 16, and 32 dSm^-1^, roughly reflecting the range of salinities encountered over time in the Doñana marsh where seeds were collected (Espinar and Serrano, 2009; Paredes et al., 2021).

These six salinities were used to store seeds for three different exposure times of 1, 6, and 12 months (i.e. 30, 180, and 365 days). Both the ingested seeds (passage) and non-ingested seeds (controls) were randomly distributed into groups of 25 seeds. For each salinity × time treatment, four groups (each placed in a separate 100 ml flask) were used for control seeds and another four for passage seeds. In total, this represented 1800 control seeds and 1800 passage seeds, for each of the three plant species. During the exposure period, the flasks were stored in the dark at 4°C (stratification conditions, after Clevering, 1995). In addition, further sets of passage and control seeds (four groups of 25 for each) were immediately placed to germinate without storage in the saline solutions (note, these seeds were some of those included in previous studies by Espinar et al., 2004, Espinar et al., 2006, which did not incorporate simulated seed banks).

### Germination

No germinations occurred during cold storage. Once the exposure time (i.e. simulated time in the seed bank) had elapsed, each group of seeds was washed with demineralized water and placed in an individual Petri dish containing a Whatman N° 1 filter paper and 20 ml demineralised water. Salinity varies greatly within and between years in the Doñana marsh, which is flushed with freshwater during major rainfall events (Espinar & Serrano, 2009; Paredes et al., 2021). Since *S. litoralis* seeds only germinate readily if they are submerged (Espinar et al., 2005a), 250 ml precipitation flasks were used for this species instead of Petri dishes, so as to keep the seeds continuously under water. Dishes and flasks containing seeds were placed in a germination chamber, with a 12h/12h light-darkness photoperiod and a light intensity of 200 μmol photons m^-2^, and temperature cycles of 25°C/10°C. Every two days for one month, the number of seeds germinated in each dish/flask was observed. We present germinability results based on the proportion of seeds that had germinated after 30 days. For each group of 25 seeds, we calculated the number of days taken for half of those seeds that germinated by the end of the experiment to germinate (T50%), as an estimate of the speed of germination (after Espinar et al., 2004, Espinar et al., 2006). In other words, T50% represents the median time to germination for each group, excluding seeds which did not germinate within 30 days.

### Data analysis

We conducted statistical analyses on seeds subjected to storage in solutions of different salinities, analyzing the effects of exposure time, salinity, and gut passage simultaneously using generalized lineal models (GLMs). We did not include the set of seeds that were not placed in storage solutions, since there were no salinity treatments comparable to those for other seeds. Furthermore, these “zero time seeds” were previously analyzed by Espinar et al. (2004; Espinar et al., 2006). Espinar et al., 2004 analyzed the effect of gut passage on immediate seed germination across a salinity gradient from 0 to 32 dSm^-1^. Furthermore, Espinar et al., 2006 analyzed the effect of seed storage conditions (salinity from 0 to 32 dSm^-1^ and exposure time) on germination patterns without gut passage.

In our GLMs, the different germination parameters (germinability, and germination time) were response variables, with duck treatment (passage or control), salinity, and storage/exposure time as predictors. With germinability (i.e. whether a seed germinated or not) as the dependent variable, we used a Binomial error distribution with logit function. With germination time (T50%) as the dependent variable, we used a Poisson error distribution with a log-link function (Dobson, 2002). Models were corrected for over-dispersion. We used conductivity and conductivity squared as continuous predictors, to allow for linear and non-linear effects of the salinity gradient. Conductivity was first log-transformed, to remove heteroscedasticity. Time was included as a categorical predictor, and we included the first order interactions between time and salinity. As expected from previous studies (Espinar et al., 2004, Espinar et al., 2006), when duck treatment was included as a categorical predictor in provisional GLMs, there was a strong effect of gut passage in all three plant species, and passage also had strong interactions with other predictors (results not shown). Therefore, in order to facilitate interpretation of the effects of salinity and exposure time, we present separate GLM analyses for seeds from gut passage and control treatments, and for each plant species.

The effects of predictors were tested using Wald tests (Dobson, 2002). Akaike’s information criterion (AIC) was used as a guide to select the best models (i.e., those being more parsimonious and with a better fit, minimizing the AIC values). All data analyses were performed using the Statistica 6.0 software package (Statsoft, 2001).

## Results

### Germinability in response to gut passage, salinity, and seed storage time

According to GLMs, the Time of exposure to the saline storage solution and a Time × Salinity interaction were both important in explaining germinability of non-ingested control seeds for all three plant species, with no evidence of curvilinear relations between germinability and salinity (**Table 1**). Seeds that had passed through the avian gut showed a germination response that was significantly different to that of control seeds, but in a manner specific to plant species. Only Time was selected in the final model for *J. subulatus* after passage. Curvilinear effects of Salinity were evident for *S. litoralis* and *B. maritimus* after passage. These were combined with a Time effect for *S. litoralis*, and a Time x Salinity interaction for *B. maritimus* (**Table 1**). Germinability of *S. litoralis* after passage peaked at intermediate salinities. In *B. maritimus* after passage, germinability peaked at intermediate salinities after 365 days exposure, but at the highest salinities after shorter exposure times.

**Table 1:** Results of GLMs for germinability, with a binomial error and separate models for control seeds (C) and passage seeds (P).

Dependent variable	Effects	Level of Effect	Estimate	SE	df	WaldStat	p
Germinability							
*J. subulatus* C	Intercept		-0.85	0.054	1	240.74	0.0001
	Time				2	7.03	0.029
		30	-0.48	0.184			
		180	0.27	0.162			
	Sal × Time				2	6.07	0.048
		x30	-0.19	0.085			
		x180	0.14	0.073			
*J. subulatus* P	Intercept		0.369	0.048	1	58.10	0.0001
	Time				2	34.87	0.0001
		30	-0.336	0.067			
		180	-0.033	0.068			
*B. maritimus* C	Intercept		-0.88	0.265	1	11.13	0.0008
	Time				2	13.06	0.001
		30	-0.54	0.182			
		180	0.56	0.170			
	Sal × Time				2	13.49	0.001
		x30	0.11	0.080			
		x180	-0.282	0.079			
*B. maritimus* P	Intercept		-1.68	0.267	1	11.13	0.0008
	Sal		0.90	0.291	1	9.52	0.002
	Sal^2^		-0.21	0.068	1	9.50	0.002
	Sal × Time				2	2.08	0.35
*S. litoralis* C	Intercept		-0.54	0.049	1	123.85	0.0001
	Time				2	19.53	0.0001
		30	-0.20	0.160			
		180	0.67	0.156			
	Sal				1	0.25	0.62
	Sal × Time				2	17.27	0.0001
		x30	0.03	0.072			
		x180	-0.27	0.071			
*S. litoralis* P	Intercept		1.59	0.295	1	29.32	0.0001
	Time				2	53.41	0.0001
		30	-0.58	0.191	1		
		180	-1.27	0.190			
	Salinity		-0.70	0.306	1	5.26	0.022
	Sal^2^		0.13	0.071	1	3.47	0.062

We present the best models selected by AIC. Exposure time of 365 days was aliased in the models, and so effectively had an estimate of zero. Sal^2^, salinity squared.

**Figure 1:**
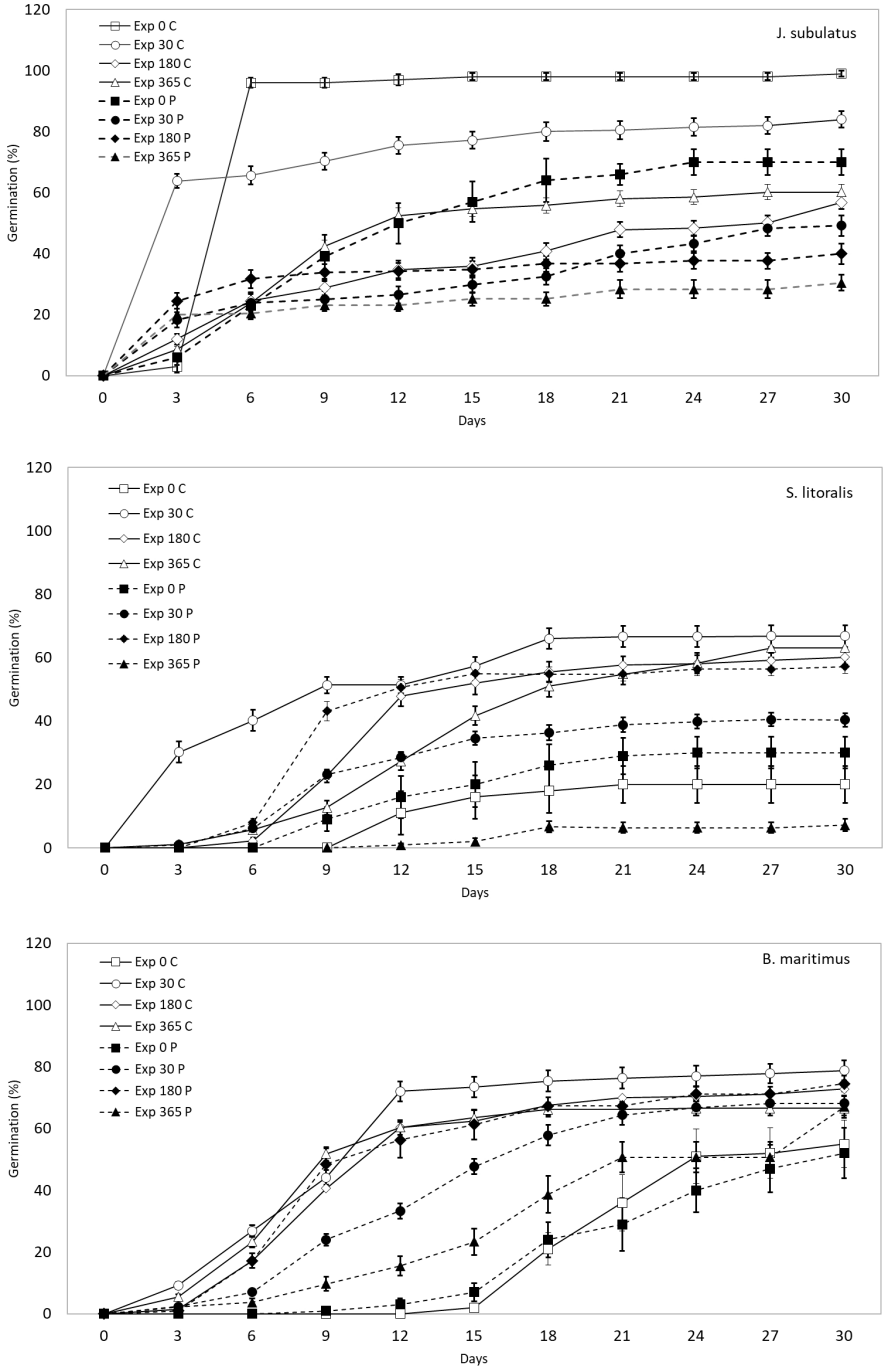
The proportion of control (C) and passage (P) seeds that germinated over time within 30 days, for different plant species and exposure times in saline storage solutions (0, 30, 180 and 365 days).

**Figure 2:**
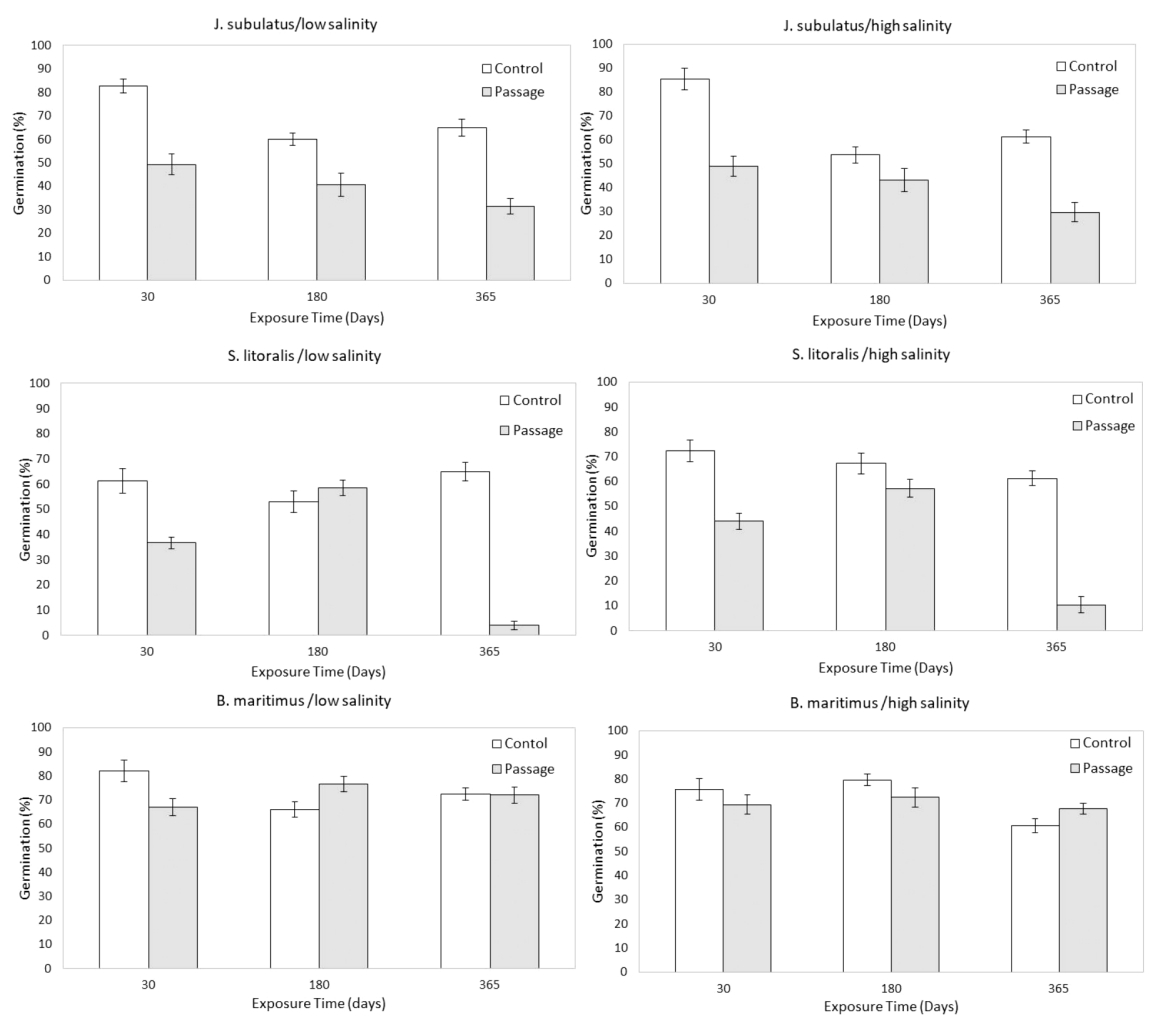
Germinability of control (C) and passage (P) seeds at different exposure times and salinities, pooled by low (1, 2, 4 dSm^-1^) and high (8, 16, and 32 dSm^-1^) conductivities for storage solutions. Means ± SE are represented. .

Germinability was consistently lower for passage than for control seeds for *J. subulatus*, and was lower for passage seeds in *S. litoralis* except for low salinities at 180 days exposure (**Figures 1**, **2**). Germinability was not consistently reduced by gut passage in *B. maritimus*, although passage changed the response to salinity to some extent (**Figure 2**). In particular, after 180 days, germinability of *B. maritimus* was highest for control seeds at high salinities, but after passage at low salinities (**Figure 2**).

Germinability was highest at minimum exposure times (30 days) for control seeds and passage seeds of *J. subulatus* (**Figures 1**, **2**). Germinability of passage seeds of *B. maritimus* and *S. litoralis* was highest at intermediate exposure times (180 days, **Figure 2**). For control seeds of *B. maritimus*, germinability was increased at higher salinities after 180 days exposure, whilst for control seeds of *S. litoralis* this occurred after both 30 and 180 days (**Figure 2**). In *S. litoralis*, there was a particularly strong reduction of germinability after 365 days after gut passage (**Figures 1**, **2**). For seeds that had passed through the avian gut, after 365 days of storage in a saline solution, overall germinability remained at 67% for *B. maritimus*, 30% for *J. subulatus*, and 7% for *S. litoralis* (**Figure 1**).

### Time to germination in response to gut passage, salinity, and seed storage time

**Figure 3:**
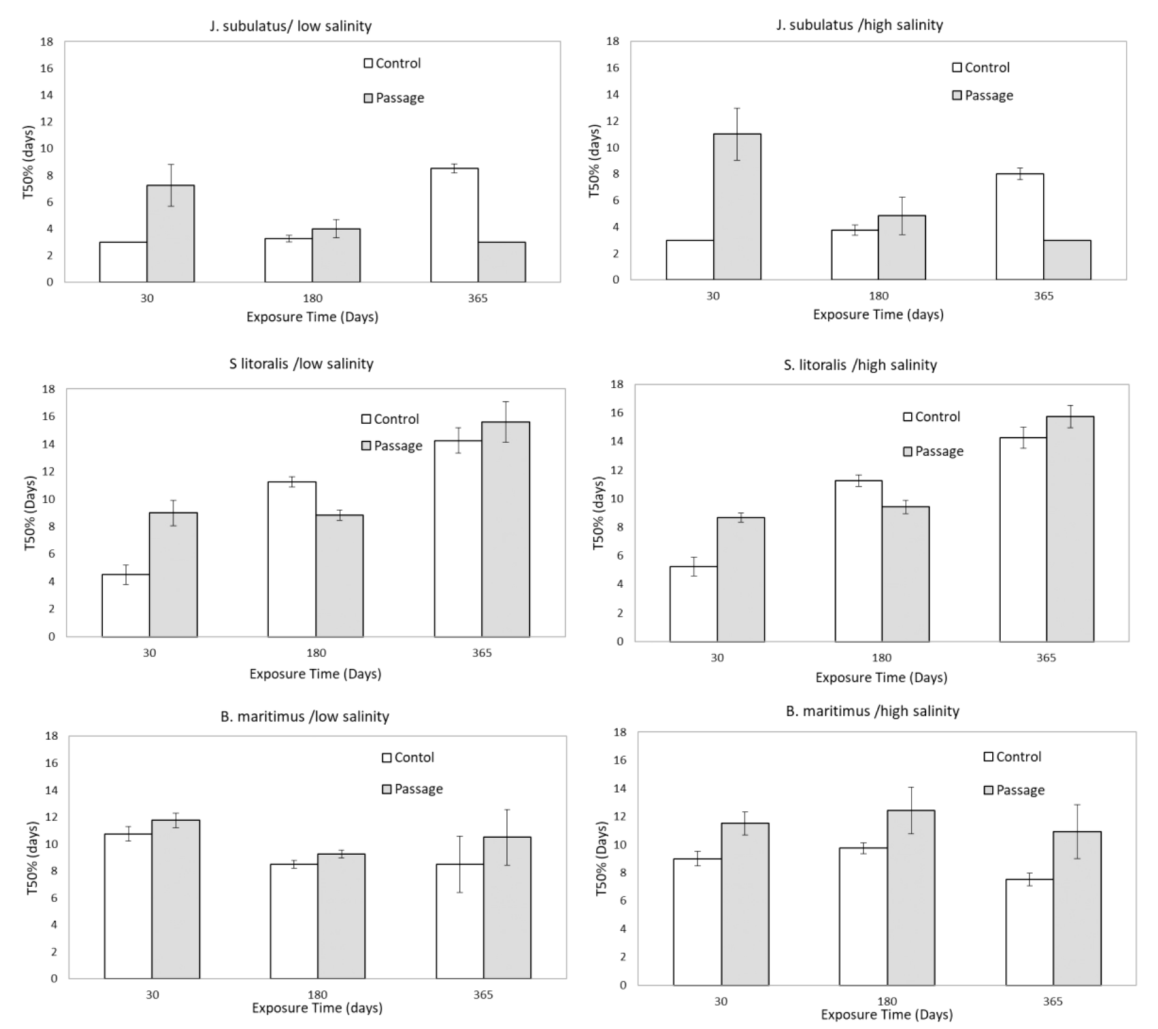
Median time to germination of control (C) and passage (P) seeds at different exposure times and salinities, pooled by low (1, 2, 4 dSm^-1^) and high (8, 16, and 32 dSm^-1^) conductivities for storage solutions. Means ± SE are represented.

For seeds that germinated, gut passage had a strong effect on median time to germination (T50%), but in a species-specific manner (**Figures 1**, **3**). Passage seeds of *J. subulatus* germinated more slowly than control seeds after 30 days exposure, but faster than control seeds after 365 days. Passage seeds of *B. maritimus* germinated consistently more slowly than control seeds. Passage seeds of *S. litoralis* germinated faster than control seeds after 180 days, but more slowly after 30 or 365 days (**Figures 1**, **3**).

GLMs revealed that T50% was consistently influenced by Time of exposure in all species (**Table 2**). Salinity x Time interactions were also important in *J. subulatus* (but only after gut passage) and *B. maritimus* (**Table 2**). Curvilinear effects of salinity were apparent in *B. maritimus*, but only following gut passage (**Table 2**) when T50% was shortest at intermediate salinities in *B. maritimus*, but only for exposures of 180 or 365 days (there was no effect of salinity after 30 days). For passage seeds, germination was notably slower at higher than at lower salinities in *J. subulatus* after 30 days, and in *B. maritimus* after 180 days (**Figure 3**).

**Table 2:** Results of GLMs for germination time, with a Poisson distribution and a log-link function, and separate models for control seeds (C) and passage seeds (P).

Dependent variable	Effects	Level of Effect	Estimate	SE	df	WaldStat	p
T50%							
*J. subulatus* C	Intercept		1.55	0.269	1	33.25	0.0001
	Time				2	19.79	0.0001
		30	1.55	0.269			
		180	-0.34	0.203			
*J. subulatus* P	Intercept		1.23	0.126	1	95.00	0.0001
	Sal		0.16	0.055	1	9.21	0.0024
	Sal × Time				2	83.34	0.0001
		x30	0.27	0.029			
		x180	-0.04	0.034			
*B. maritimus* C	Intercept		1.94	0.197	1	96.61	0.0001
	Time				2	5.77	0.056
		30	0.27	0.125			
		180	-0.25	0.131			
	Sal × Time				2	5.59	0.061
		x3	-0.08	0.058			
		x180	0.13	0.058			
*B. maritimus* P	Intercept		3.32	0.169	1	383.7	0.0001
	Time				2	8.43	0.015
		30	0.23	0.106			
		180	-0.30	0.113			
	Sal		-1.27	0.195	1	42.54	0.0001
	Sal^2^		0.32	0.045	1	49.55	0.0001
	Sal × Time				2	8.93	0.011
		(x30	-0.08	0.046			
		x180	0.13	0.047			
*S. litoralis* C	Intercept		1.99	0.192	1	107.49	0.0001
	Time				2	28.54	0.0001
		30	-0.818	0.160			
		180	0.285	0.125			
*S. litoralis* P	Intercept		2.381	0.039	1	3596.6	0.0001
	Time				2	6.90	0.032
		30	-0.202	0.056			
		180	-0.170	0.055			

We present the best models selected by AIC. Exposure time of 365 days was aliased in the models, and so effectively had an estimate of zero. Sal^2^, salinity squared.

### Comparison with seeds placed to germinate immediately

Both for germinability and germination time, the effects of storage in saline solutions for 30-365 days, compared to no storage at all, were complex and variable between plant species and duck treatment (**Figure 1**). For *B. maritimus*, storage consistently increased both germinability and speed of germination, for both control and passage seeds. For *S. litoralis*, storage generally increased germinability and speed of germination, with the exception of passage seeds stored for 365 days, which performed worse than unstored seeds. For *J. subulatus*, storage consistently reduced germinability, although it increased the proportion of seeds germinating within 3 days (**Figure 1**).

## Discussion

We found strong evidence that seeds from wetland plants can remain viable within seed banks after endozoochory by waterbirds. After gut passage, seeds of three species of sedges and rushes could be stored in the dark (as if buried in inundated sediments) and at a range of salinities that reflect field conditions, for up to 12 months before germination. Subsequent germination was modified by gut passage, in comparison with non-ingested control seeds, in a manner that depended on both the time that seeds were stored in cold water (simulating presence in a seed bank), and the salinity of that water. Furthermore, both for germinability and for speed of germination, the direction and strength of time and salinity effects varied between plant species, as did time x salinity interactions.

Previous reviews of endozoochory by frugivores and herbivorous mammals have shown that the effects of gut passage on germinability and germination time vary considerably between plant species and studies (Jaganathan et al., 2016; Soltani et al., 2018), but much less information is available for waterbirds. Waterbird endozoochory is especially frequent in coastal ecosystems where migratory waterbirds are particularly abundant (MWO, 2018), and where wetland plants are generally halotolerant. The number of waterfowl (Anatidae: ducks, geese and swans) species known to disperse a given plant species is positively correlated with Ellenberg indicators of halotolerance, as well as with increasing moisture requirements (Almeida et al., 2022). If seeds carried by waterbirds can enter seed banks, this will facilitate the role of these dispersal vectors in plant meta-population dynamics and gene flow (Tomowski et al., 2023). After endozoochory, seeds do not need to become established immediately, but can remain in the seed bank until suitable conditions arise. For example, seeds arriving at a site during autumn migration may readily enter the seed bank before germinating the following spring. Waterfowl endozoochory of aquatic plant seeds is likely to be more frequent during autumn migration than in spring (Urgyán et al., 2023, but see Figuerola et al., 2003). Avian gut passage reduces floatability of wetland plant seeds (authors, unpublished data), probably due to increased water permeability following damage to the pericarp and seed coat incurred during passage (Clevering, 1995; Costea et al., 2019). Therefore, when faeces are egested into water following endozoochory, seeds are likely to sink and be incorporated into seed banks in the sediments. Genetic studies of aquatic seed banks may detect migration events via zoochory (Tomowski et al., 2023). Population genetic studies support the role of waterfowl in long-distance dispersal of sedges (Kettenring et al., 2019).

*Schoenoplectus litoralis* grows at greater depths than the other two species studied (Espinar et al., 2005b). It showed a particularly positive response to gut passage after storage for 180 days, when it had a higher germinability than after 30 or 365 days, and a faster germination than control seeds. After gut passage, *B. maritimus* also had the highest germinability after 180 days. These responses could potentially be adaptive, if seeds first arrive by endozoochory to a dry microhabitat or to a wetland during autumn migration, and germinate the following spring. In Doñana, habitat suitable for this species typically floods 3–5 months before germination occurs in spring (Green et al., 2017). High rates of waterfowl endozoochory have been recorded in the field for both these Cyperaceae species, in contrast to *J. subulatus* (Espinar et al., 2006; Almeida et al., 2022; Urgyán et al., 2023). Our results for germination patterns in three species are consistent with van Leeuwen et al. (2023) who found that, following prior stratification, simulated gut passage was more likely to increase germinability and reduce germination time for plant species with higher Ellenberg moisture requirements. Of our study species, *J. subulatus* has the lowest moisture requirements and was the one we found to have the fewest positive effects of gut passage on germination.

Previous studies may have underestimated the ability of aquatic seeds to incorporate into seed banks after gut passage. For example, Tol et al., 2021 concluded that seeds of the seagrass *Zostera muelleri* are unlikely to enter the seed bank after gut passage by marine herbivores (green sea turtles and dugongs), because after a germination test of 60 days at 19-32°C, no seeds remained viable according to a tetrazolium test. However, if they had not been placed at those temperatures, they may have retained viability for much longer (Infante-Izquierdo et al., 2023). Our results suggest that aquatic seeds in general have the capacity to enter seed banks for extended periods if they are not initially exposed to necessary germination cues (e.g. an increase in light intensity, or a reduction in salinity). After endozoochory of plants lacking a fleshy fruit by ungulates and other mammalian herbivores, seeds often enter seedbanks in terrestrial habitats such as grasslands and forests (Malo et al., 1995; Pakeman et al., 1999; Jaroszewicz, 2013). Non-classical endozoochory by ungulates and waterfowl has many similarities (Green et al., 2022), and the role of both these vector groups in contributing to seedbanks is likely to be one of them.

### Salinity effects

The majority of studies addressing the impact of vertebrate gut passage on germination conducted immediate germination tests at near-zero salinity (including those on frugivores and herbivores; Jaganathan et al., 2016; Soltani et al., 2018), without considering the potential importance of soil or water salinity in the field. Salinity is a critical environmental gradient in all coastal wetlands and estuaries, as well as inland wetlands and soils in general in Mediterranean, semi-arid, and arid climates. Increases in salinization due to global heating, sea level rise, and increases in water abstraction pose a major threat to wetland biodiversity (Taylor et al., 2021; Cunillera-Montcusí et al., 2022).

We demonstrated that the effect of gut passage on germination response of sedges and rushes along a salinity gradient is complex and species-specific. Our work builds on previous studies using seeds from the same plant populations (Espinar et al., 2004; Espinar et al., 2006) which showed that germination responses immediately after gut passage, and their relation with the response of control seeds, depend on the salinity at which a germination test is conducted. For *S. litoralis*, gut passage increased germinability and the speed of immediate germination at low salinities of 0-2 dSm^-1^, whereas the opposite occurred at salinities of ≥4 dSm^-1^ (Espinar et al., 2004). In our study, the germination test itself was conducted with demineralized water. Freshwater pulses are known germination cues for plants in coastal wetlands (Muñoz-Rodríguez et al., 2017; Castillo et al., 2021; Tol et al., 2021), and removal from saline solution into fresh germination conditions may well be a germination cue for our sedge and rush species. Furthermore, the salinity at which seeds were stored before our germination tests had a strong influence on the germination response of both control and gut passage seeds, as well as on the difference between them. Given the mechanical effects of gut passage on seed architecture (Costea et al., 2019), it is not surprising that there were important differences with control seeds in the response to storage in saline solution. Nevertheless, we did not find consistent evidence that higher salinities reduce the germinability of seeds after gut passage, despite the resulting increase in permeability of the seed coat.

Our results illustrate the complexity of how avian gut passage affects the germination response of wetland plants. A caveat for our study is that, since many seeds are destroyed during gut passage by digestive processes, it is possible that seeds recovered after gut passage are partly selected by this process. Hence, recovered seeds may not be fully representative of ingested seeds, complicating their comparison with control seeds. Brochet et al. (2010) found that gut passage may increase the proportion of viable seeds by selective digestion of non-viable ones. Ideally, we should have tested the viability of all the seeds in our study that did not germinate within 30 days with a tetrazolium test, to separate seeds that were dead from those that remained dormant. This limitation does not apply to studies simulating digestive processes with laboratory protocols (van Leeuwen et al., 2023), in which the fate of each individual seed can be monitored.

### Dormancy strategies

We stored seeds in darkness at 4 °C between their collection and starting the feeding experiment, and again during seed bank simulation, with the aim of breaking physiological dormancy to assure high germination rates (Clevering, 1995). However, we still found evidence that gut passage had an additional effect in breaking dormancy for sedges in some treatments (i.e. germinability was often higher than for control seeds after 180 or 365 days of storage, **Figure 2**). Thus, our initial hypothesis, based on dormancy classification (Baskin & Baskin, 2004), that gut passage would always inhibit germination, was rejected. Our results bring into question the assumptions used to classify dormancy strategies (see also Costea et al., 2019; van Leeuwen et al., 2023), and underlines both the importance of endozoochory and the complexity of dormancy for plants of dynamic wetland ecosystems (see also Marty and Kettenring, 2017).

Both passage through the waterfowl gut, and cold storage, may act to break dormancy in sedges. *Schoenoplectus lacustris* and *B. maritimus* can persist in seed banks for many years (Clevering, 1995), and it is possible that the increases in germinability we observed after gut passage may have disappeared if we had used much longer storage periods. However, Clevering (1995) argued that germination in these species is mainly triggered by permeability of the seed coat, rather than some internal clock, and gut passage can provide that trigger.

Temperature is a critical variable influencing dormancy and germination responses, and in our study area with a Mediterranean climate a temperature as low as 4 °C is only experienced in winter months (Espinar et al., 2015). Hence, our results may have changed if seeds had been stored under different temperature regimes, and our study only represents a first approximation of what might occur in a natural wetland when seed banks are inundated over periods of up to 12 months.

## Conclusions

Our experiment simulated the presence of seeds in a seed bank in inundated sediments in a general manner, and suggests that waterfowl endozoochory can lead to effective dispersal even when seeds are moved by birds into microhabitats where conditions are not immediately suitable for germination. The seeds can remain for weeks, months, or at least a year in the seed bank until conditions become suitable. Research into establishment success of plants after waterbird endozoochory events has been highlighted as a future priority (Green et al., 2023), and our study represents an important step which supports the potential for establishment long after endozoochory has occurred. Further studies (e.g. with mesocosms) should address the fate and fitness of seeds incorporated into seedbanks after waterfowl endozoochory.

## Data availability statement

The original contributions presented in the study are included in the article/**Supplementary material**. Further inquiries can be directed to the corresponding author.

## Ethics statement

Ethical approval was not required for the study involving animals in accordance with the local legislation and institutional requirements because the experiment was conducted in 2002, before such a requirement existed in Spain (as described in the manuscript).

## Author contributions

JE: Conceptualization, Data curation, Formal Analysis, Investigation, Methodology, Writing – review & editing. JF: Conceptualization, Investigation, Methodology, Writing – review & editing. AG: Conceptualization, Funding acquisition, Methodology, Project administration, Writing – original draft.
